# Methylation of phase II metabolites of endogenous anabolic androgenic steroids to improve analytical performance

**DOI:** 10.1002/dta.3694

**Published:** 2024-04-21

**Authors:** Sandra Pfeffer, Guenter Gmeiner, Guro Forsdahl

**Affiliations:** ^1^ Department of Pharmacy UiT – The Arctic University of Norway Tromsø Norway; ^2^ Doping Control Laboratory Seibersdorf Labor GmbH Seibersdorf Austria

**Keywords:** derivatization, doping, endogenous steroids, methylation, phase II metabolites

## Abstract

The study of intact phase II metabolites of endogenous anabolic androgenic steroids (EAAS) gives important information about metabolism and has the potential to improve the detection of doping with testosterone. For analysis with liquid chromatography–mass spectrometry (LC‐MS), chemical derivatization at the steroid moiety is a technique to improve the positive ionization efficiency of glucuronidated/sulfated EAAS under collision‐induced dissociation (CID) conditions. However, regarding the chromatographic performance, there are still challenges to address, for example, poor peak shape, which is mainly caused by nondefined adsorption in the chromatographic system. Here, we show a novel derivatization technique for the analysis of selected phase II metabolites of EAAS, where the acidic moiety of the glucuronide/sulfate is methylated with different methylation reagents to reduce nondefined adsorption. The methylation reagent trimethylsilyl‐diazomethane (TMSD) was preferred over the other tested reagents methyl iodide (MeI) and dimethyl sulfate (DMS). Glucuronidated and sulfated testosterone and epitestosterone were methylated, and their chromatographic performance and CID ion mass spectra obtained in positive ionization mode were investigated. The peak width and peak height were significantly improved for all substances. Methylated testosterone sulfate showed the best results with a 3.5 times narrower peak and 14 times increased intensity compared with underivatized testosterone sulfate. Furthermore, CID ion mass spectra obtained in positive ionization mode showed product ions characteristically for the steroidal backbone for all substances. This preliminary study shows the potential of methylation as a supplementary derivatization technique, which can assist in the development of more sensitive methods due to the improvements in method performance.

## Graphical abstract

Intact phase II metabolites of endogenous anabolic androgenic steroids give important information about metabolism and have the potential to improve the detection of doping with testosterone. Currently applied methods for liquid chromatography–mass spectrometry analysis include chemical derivatization at the steroid moiety, which can pose challenges regarding chromatographic performance, for example, poor peak shape. Methylation with trimethylsilyl‐diazomethane at the acidic moiety of the phase II conjugate improves analytical performance by the formation of narrower and more intense peaks.

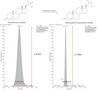

## 1 INTRODUCTION

Anabolic agents are used by athletes to improve their sports performance by generating enhanced muscle growth and strength. Adverse health risks are well known and include severe long‐term effects such as the increased risk of cancer, cardiac hypertrophy, liver dysfunction, and masculinization in women.^1^, ^2^ Because anabolic agents pose these severe health risks and can enhance sports performance, they have been added to the list of prohibited substances in sports. They comprise synthetic anabolic androgenic steroids (AAS) and exogenously administered endogenous anabolic androgenic steroids (EAAS).^3^ Currently, substances from the group of anabolic agents are responsible for more than a third of all reported positive findings by doping control laboratories accredited by the World Anti‐Doping Agency (WADA).^4^ The detection of doping with endogenous anabolic agents constitutes an ongoing challenge because of short detection windows and difficulties in proving EAAS misuse due to their natural presence in the human body. Hence, improvement of current and development of new analytical methods for the detection of doping with EAAS is required.^5^

The identification of anabolic agents in doping control laboratories is accomplished by the detection of target substances and their metabolites in urine samples. The excretion of AAS and EAAS in urine follows extensive phase I and II metabolism in the human body. Oxidation, reduction, or hydroxylation reactions take place in phase I metabolism to increase polarity. Additional conjugation reactions to form glucuronides or sulfates in phase II metabolism increase polarity further and enable the elimination via renal routes.^6^ Detection of AAS and EAAS in routine doping analysis is based on selective hydrolysis of glucuronide metabolites in urine, resulting in the measurement of free, unconjugated steroids. These are analyzed after derivatization mainly by gas chromatography‐tandem mass spectrometry (GC‐MS/MS).^7^, ^8^ This procedure leads to the loss of important information concerning phase II metabolites, especially sulfate‐conjugated metabolites. During the last years, interest in direct analysis of phase II metabolites of AAS and EAAS has risen, revealing important insights into metabolism and alterations in endogenous steroid metabolism after testosterone doping.^5^

Liquid chromatography–mass spectrometry (LC‐MS) and liquid chromatography–high resolution mass spectrometry (LC‐HRMS) analysis of phase II metabolites of AAS and EAAS has been described in numerous reports. To gain interesting insights into the metabolism of AAS, direct‐injection and online solid phase extraction (SPE) methods for LC‐MS have been used instead of the time‐consuming sample preparation needed for conventional methods. Furthermore, it has been demonstrated that phase II metabolites of AAS can expand the detection window for certain substances.^9^, ^10^, ^11^, ^12^ The quantification of glucuronidated and sulfated EAAS in urine and serum has revealed novel information about metabolism, which can be used to significantly improve the current monitoring model. The potential of using phase II metabolites of EAAS as biomarkers to detect misuse of testosterone has been demonstrated.^13^, ^14^, ^15^, ^16^

In general, different methods have been developed to improve the detection of AAS and EAAS phase I metabolites by LC‐MS.^5^ Ionization efficiency can be a limitation for sensitive methods, depending on the proton affinity of the steroid.^17^ Different methods of chemical derivatization have been investigated to improve electrospray ionization (ESI) efficiency in LC‐MS analysis of unconjugated steroids.^18^, ^19^, ^20^, ^21^, ^22^ Briefly summarizing these methods, easily ionizable or permanently charged moieties are attached to the molecule, leading to better ionization in positive ESI mode and hence more sensitive methods. Analytical procedures for the characterization of intact phase II metabolites of EAAS have mainly been carried out with LC‐MS in negative ESI mode because positive ESI mode provides low sensitivity due to poor ionization for certain substances.^23^ Despite high ionization efficiency in negative ESI mode, poor ion mass spectra are obtained under collision‐induced fragmentation (CID) conditions. Obtained fragments representing the sulfate/glucuronide moiety lack structural information and are therefore not ideal for identification. To overcome these problems, chemical derivatization techniques established for the analysis of AAS and EAAS phase I metabolites have also been applied to phase II metabolites of EAAS.^24^, ^25^ It has been reported that chemical derivatization of EAAS phase II metabolites enhances ionization efficiency in positive ESI mode and therefore leads to more characteristic fragmentation patterns in CID. Nevertheless, there are still challenges regarding chromatography to overcome. Acidic moieties in conjugated steroids, especially in sulfates, often lead to poor peak shape (tailing, broad peaks) caused by nondefined adsorption in the chromatographic system.^26^ Also, chemical derivatization of certain ketosteroids with, for example, Girard reagents, leads to the formation of E‐/Z‐isomers resulting in two peaks, which can lead to coelution with isobaric compounds, a more complicated quantification, and cause lower sensitivity.^24^, ^27^

This project aimed to improve the analytical performance regarding the analysis of phase II metabolites of EAAS. A novel derivatization technique was investigated, which not only enhances ionization efficiency in positive ESI mode but also improves chromatographic performance. To reduce nondefined adsorption and enhance ionization efficiency in positive ESI mode, methylation of the acidic moieties of glucuronides and sulfates was investigated. In literature, methylation has been applied to reduce the polarity of substances for GC‐MS analysis^28^, ^29^ or to prepare highly polar compounds, for example, substances containing phosphate groups, for LC‐MS.^30^ In this work, methylation was tested upon the model compounds testosterone sulfate (TS), epitestosterone sulfate (ES), testosterone glucuronide (TG), and epitestosterone glucuronide (EG). These exemplary compounds were chosen to represent EAAS, which tend to appear in low concentration ranges and therefore would benefit most from improved analytical performance. After testing the three derivatization reagents methyl iodide (MeI), dimethyl sulfate (DMS), and trimethylsilyl‐diazomethane (TMSD), the most suitable reagent TMSD was applied to urine samples to confirm the suitability of methylation for quantification of EAAS phase II metabolites.

## 2 MATERIALS AND METHODS

### 2.1 Chemicals, reagents, and materials

Conjugated steroids, as well as deuterated conjugated steroids used as internal standards (IS), were purchased from National Measurement Institute Australia (Sydney, Australia). Stock solutions with a concentration of 1 mg/mL for IS and standard substances were prepared by dissolving 1 mg of standard substance in 1 mL of methanol. Individual or mixed working solutions at appropriate concentrations were prepared by diluting stock solutions with methanol and stored at −20°C. The IS mix was prepared with the following concentrations: 4 μg/mL D_3_‐TG, 4 μg/mL D_4_‐EG, 1 μg/mL D_3_‐TS, and 1 μg/mL D_3_‐ES.

Methanol (HPLC grade) for standard solutions and sample preparation was supplied by Chem‐Lab (Zedelgem, Belgium). Water used for sample preparation was provided by a Milli‐Q water purification system (Millipore, Reference A+, Burlington, MA, USA). Acetonitrile (HPLC grade) for sample preparation was bought from Merck (Darmstadt, Germany). Water and acetonitrile (ULC/MS‐CC/SFC grade), as well as formic acid (99%, ULC/MS‐CC/SFC grade) used for HPLC analysis, were purchased from Biosolve BV (Valkenswaard, Netherlands).

Derivatization reagents dimethyl sulfate (99%+, AcroSeal) and trimethylsilyl‐diazomethane (2‐M solution in hexane, Acros Organics) were bought from Thermo Fisher Scientific (Waltham, MA, USA). The derivatization reagent methyl iodide (purum, ≥99.0%) and potassium carbonate used for derivatization experiments were purchased from Merck (Darmstadt, Germany).

Potassium dihydrogen phosphate, sodium chloride, disodium hydrogen phosphate, and ammonium chloride were obtained from Merck (Darmstadt, Germany). Urea was provided by GE Healthcare Life Sciences (Uppsala, Sweden), and creatinine was purchased from Sigma Aldrich (St. Louis, MI, USA). SPE Oasis HLB cartridges (60 mg/3 mL, 30‐μm particle size) were obtained from Waters (Milford, MA, USA).

### 2.2 Sample preparation

#### 2.2.1 Derivatization

To select the optimal derivatization technique, three different derivatization reagents, namely, methyl iodide (MeI), dimethyl sulfate (DMS), and trimethylsilyl‐diazomethane (TMSD), were tested using the following derivatization protocols:

##### MeI

In a test tube, 5 μL of a standard solution containing all four target compounds and IS (1 mg/mL in methanol) were transferred and evaporated to dryness. The dry extract was reconstituted in 170 μL acetonitrile. Approximately 20 mg of potassium carbonate and 30 μL of the derivatization reagent MeI were added. The mixture was incubated at 60°C for 3 h; 100 μL of the supernatant was mixed with 50 μL water and analyzed by LC‐HRMS.^29^, ^31^

##### DMS

In a test tube, 5 μL of a standard solution containing all four target compounds and IS (1 mg/mL in methanol) were transferred and evaporated to dryness. The dry extract was reconstituted in 100 μL acetonitrile. Approximately 20 mg of potassium carbonate was added; 20‐, 50‐, or 100 μL derivatization reagent DMS was added. The mixtures were incubated at ambient temperatures for 2 h, 5 h, or overnight; 100 μL of the supernatant was mixed with 50 μL water and analyzed by LC‐HRMS.^32^

##### TMSD

In a test tube, 5 μL of a standard solution containing all four target compounds and IS (1 mg/mL in methanol) were transferred and evaporated to dryness. The dry extract was reconstituted in 100 μL of a mixture of acetonitrile/methanol: 1/1 (v/v). Volumes 20 , 50 , or 100 μL derivatization reagent TMSD were added. The mixtures were incubated at ambient temperatures for 2 h, 5 h, or overnight. The mixture was evaporated to dryness, reconstituted in 100 μL water/acetonitrile: 1/1 (v/v), and analyzed by LC‐HRMS.^28^, ^33^, ^34^, ^35^

#### 2.2.2 Preparation of artificial urine

Artificial urine was used as a matrix for testing method performance and was prepared as follows: 2.5 g/L potassium dihydrogen phosphate, 9.0 g/L sodium chloride, 2.5 g/L di‐sodium hydrogen phosphate, 3.0 g/L ammonium chloride, 25 g/L urea, and 2 g/L creatinine were prepared in water.

#### 2.2.3 Solid phase extraction

Extraction was performed on Oasis HLB cartridges (60 mg, 30‐μm particle size) as follows: cartridges were conditioned with 2 mL of methanol and equilibrated with 2 mL of water. The loading solution was prepared by the addition of 25 μL of IS solution to 2 mL of urine and loaded onto the cartridges, followed by washing with 2 mL of water. Finally, the analytes were eluted with 2 mL of methanol. The eluate was evaporated to dryness, and the dry extract was reconstituted in 100 μL of a mixture of acetonitrile/methanol: 1/1 (v/v); 50 μL derivatization reagent TMSD were added. The mixture was incubated at ambient temperatures for 2 h, evaporated to dryness, reconstituted in 100 μL water/acetonitrile: 1/1 (v/v), and analyzed by LC‐HRMS.

#### 2.2.4 LC‐HRMS analysis

Analysis was carried out on a Q Exactive Focus Orbitrap MS analyzer coupled to a Vanquish Horizon UHPLC System (Thermo Fisher, Waltham, MA, USA). The LC system was equipped with a cooled sample tray (20°C), a high‐pressure binary pump, and a column oven set at 25°C. Chromatographic separation was carried out using an InfinityLab Poroshell 120 EC‐C18 column (100 × 2.1 mm i.d., 1.9‐μm particle size, Agilent Technologies). To reduce non‐defined adsorption, shielded fused silica nanoViper™ tubing sheathed in polyetheretherketone (PEEK) from Thermo Fisher was employed in the entire system. The mobile phase consisted of water containing 0.1% formic acid (solvent A) and acetonitrile containing 0.1% formic acid (solvent B). A gradient elution program was employed at a constant flow rate of 0.3 mL/min with solvent B increasing over 11.5 min from 10% to 98%. It was held there for 2 min before returning to 10% B within 0.1 min. The column was re‐equilibrated at 10% B for 4.4 min, resulting in a total runtime of 18 min. The injection volume was 2 μL.

The mass spectrometer was equipped with a heated electrospray ionization (HESI) source, operated in positive and negative mode (switching). Source parameters were as follows: spray voltage 3.7 kV, capillary temperature 320°C, sheath gas, and auxiliary gas (nitrogen): 30 and 10 arbitrary units, respectively. During method development, the instrument was operated in full scan (FS) mode from *m*/*z* 80–700 at 70,000 resolving power. To obtain CID ion mass spectra and for quantification purposes, the instrument was operated in parallel reaction monitoring (PRM) mode including all target substances as [M + H]^+^ ions. Collision energy (CE) was set at 25 eV, and resolving power was 17,500.

### 2.3 Method performance

#### 2.3.1 Derivatization

The peak intensity of the [M‐H]^−^ signal for the nonderivatized conjugated steroids and the [M + H]^+^ signal for the methylated conjugated steroids obtained in FS mode were used to select the optimal derivatization reagent. The reduction of the [M‐H]^−^ signal obtained from the remaining underivatized conjugated steroids was compared with a nonderivatized sample measured in the same sequence to calculate the conversion. To calculate the intensity increase the peak intensity of methylated conjugated steroids was compared with a nonderivatized sample measured in the same sequence. Formed reaction products were identified and possible side products were investigated.

#### 2.3.2 Chromatography and CID ion mass spectra

Chromatograms and ion mass spectra obtained in PRM mode were used to calculate parameters and display chromatograms and CID ion mass spectra. Chromatograms were extracted with 5‐ppm mass accuracy using the software Thermo Qual Browser. To compare the chromatography of the methylated conjugated steroids with the nonderivatized conjugated steroids, peak height and peak width at 5% peak height were measured.

#### 2.3.3 Linearity and limit of quantification

Different concentrations of standard substances were spiked into artificial urine before solid phase extraction and derivatization with TMSD were carried out. The linear range for each substance was determined by plotting the peak area ratio of the methylated conjugated steroids and the methylated deuterated internal standards at nine different concentration points. Linearity (*R* ^2^) was calculated with the software Thermo Quan Browser. The limit of quantification (LOQ) was estimated by preparing five additional concentration points in the lower linear range according to DIN 32645.^36^

#### 2.3.4 Accuracy

Eight WADA external quality assessment scheme (EQAS) samples, allocated during the last 5 years, were prepared in triplicate by solid phase extraction and derivatized with TMSD. Mean concentrations for TG and EG were calculated using the internal calibration (IC) quantification approach according to Visconti et al.^37^ The concentrations obtained were transformed into concentrations of testosterone (T) and epitestosterone (E) through molecular weight adjustment (TG, EG = 464.24 g/mol; T, E = 288.21 g/mol). Concentrations initially measured for TG and EG in ng/mL were transformed by dividing them by their respective molecular weights and then multiplied by the molecular weights of T and E to establish comparable values. These were compared with the consensus values of all laboratories participating in the EQAS rounds for T and E, and the deviation was calculated.

## 3 RESULTS AND DISCUSSION

### 3.1 Derivatization

**FIGURE 1:**
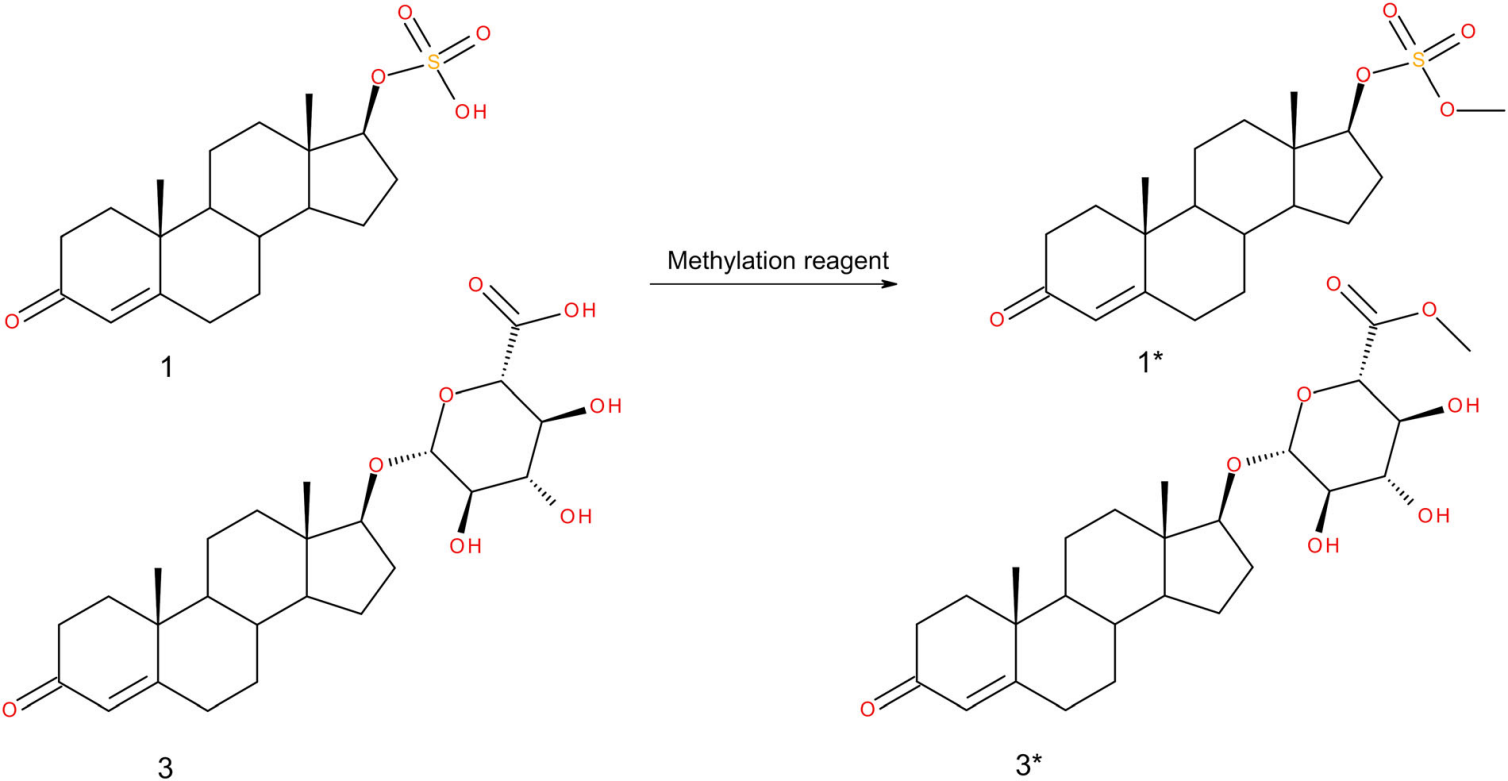
Reaction of the acidic moiety with the tested methylation reagents. TS (1) is converted to methylated TS (1*) and TG (3) is converted to methylated TG (3*).

Model compounds TS, ES, TG, and EG were methylated at the acidic moiety of the sulfonic acid or the carboxylic acid, respectively, as shown in Figure 1.

Methylation was used to obtain better peak shape, as well as positive ionization fragmentation ions under CID conditions containing structural information of the steroid structure. Three derivatization reagents (MeI, DMS, TMSD) were tested against the model compounds. For choosing the most promising derivatization reagent, MS was operated in FS mode to monitor conversion, intensity increase, and side products. MeI was eliminated from further investigation after the first measurements because the sulfonic acid moiety was not methylated, only the carboxylic acid. DMS, a stronger methylation reagent, showed adequate methylation strength for both carboxylic acids and sulfonic acids. Longer incubation time (overnight) at ambient temperatures using 20 μL DMS showed >99% conversion of all model compounds and produced derivatized compounds. Nevertheless, deconjugation of the sulfate group from the steroid took place, which was monitored by the formation of unconjugated T and E. Therefore, DMS was excluded from further testing. The derivatization reagent TMSD showed adequate methylation strength for all model compounds, and no deconjugated steroids were observed. Therefore, TMSD was chosen as the most promising methylation reagent for phase II metabolites of EAAS and was further investigated.

### 3.2 TMSD

To determine optimal reaction conditions, derivatization was carried out using 20, 50, or 100 μL of derivatization reagent and incubating for three different periods (2 h, 5 h, overnight) at ambient temperatures. In all cases, the glucuronidated steroids were methylated quantitatively as the nonderivatized steroid conjugates were detected <1% during the analysis of the derivatized samples. Sulfated steroids showed <80% conversion using 20 μL TMSD. Derivatization with 50 μL TMSD and incubation for 2 h showed >98% conversion of all model compounds. Methylated conjugated steroids were obtained with approximately 460% (TG), 220% (EG), 260% (TS), and 75% (ES) peak height compared with the underivatized conjugated steroids in FS mode. To find an explanation for the decrease of ES peak height, the FS spectrum of methylated ES was compared with the FS spectrum of underivatized ES. For both substances, deconjugation of the phase II moiety from the steroid was observed by an ion obtained at m/z 289, which corresponds to [M‐SO_3_*]^+^ and [M‐SO_3_]^+^, respectively. The signal at m/z 289 was approximately twice as high for methylated ES compared with underivatized ES, which supposes that methylation at the acidic moiety increased the destabilization of the phase II metabolite. Deconjugation could not be observed for any of the other investigated substances.

Furthermore, possible side reactions were monitored. Different from using DMS as methylation reagent, no deconjugation of the steroid and sulfate or glucuronide group was observed. No signal was obtained for the [M + H]^+^ peak of T and E. Because TMSD can methylate alcohol groups under certain reaction conditions,^38^ TG and EG were investigated regarding multiple methylation reactions. Indeed, two‐ and three‐times methylated TG and EG were observed in all samples. Multiple peaks were obtained, representing methylation at different alcohol groups of the glucuronide moiety (Figure S1, Supporting Information). The amount (peak area of all peaks) of two‐ and three‐times methylated TG and EG was stable comparing multiple derivatization experiments under the same reaction conditions and increased with longer incubation time. Incubation for 2 h showed approximately 15% side product for TG and EG, combined. An increase in multiple methylated side products in the final extracts was not observed after stopping the methylation reaction by evaporation.

Methylation with 50 μL TMSD and incubation for 2 h was chosen as optimal reaction conditions, showing sufficient conversion (>98%) and the lowest amount (<15%) of side products. After the determination of the optimal reaction conditions, CID ion mass spectra were recorded to investigate improvements in method performance.

### 3.3 Method performance

#### 3.3.1 Chromatography

Phase II metabolites of EAAS often lead to broad and tailing peaks caused by nondefined adsorption of the acidic moieties. Peak widths of >0.3 min and up to 1 min are commonly described in the literature.^14^, ^15^, ^24^ Although these methods did lead to satisfactory results, ways to improve peak shape could assist in reaching even lower LOQs (due to improved signal‐to‐noise [S/N] ratio caused by more intense peaks).

Table 1 gives an overview of the peak widths and peak heights from CID ion mass spectra of the investigated methylated compounds. Peaks obtained from methylated conjugated steroids were compared with measurements of underivatized conjugated steroids. Chromatograms were extracted for both cases, and the peak width and peak height were measured.

**TABLE 1:** Comparison of peak width and peak height of extracted chromatograms deriving from underivatized and methylated conjugated steroids.

Substance	Transitions (m/z)	Peak width (min)	Peak width improvement	Peak height	Peak height improvement
TG methylated	479.26 → 109.06	0.06	×2.8	399,226	×2
TG not derivatized	465.24 → 109.06	0.17		202,231	
EG methylated	479.26 → 109.06	0.06	×2.7	104,423	×4.5
EG not derivatized	465.24 → 109.06	0.16		23,403	
TS methylated	383.18 → 97.06	0.06	×3.5	2,181,411	×14
TS not derivatized	369.17 → 97.06	0.21		151,215	
ES methylated	383.18 → 97.06	0.08	×3	240,486	×2.8
ES not derivatized	369.17 → 97.06	0.21		87,428	

Chromatograms used for determination can be found in the supporting information (Figures S2 and S3). Transitions were selected for evaluation because they were highly abundant in methylated and not derivatized samples and showed no interference in any urine sample. Methylation of the acidic moieties found in glucuronides and sulfates led to a significant reduction of peak width, especially in sulfates. Also, for all substances, an increase in signal intensity was achieved, which can assist in developing more sensitive methods. Retention times shifted for all investigated substances. Derivatized sulfates eluted approximately 2–3 min later than the underivatized substances, where the elution order was also inverted. Derivatized TG only eluted approximately 0.3 min later than underivatized TG. Surprisingly, derivatized EG eluted approximately 2 min earlier compared with underivatized EG. The decrease in polarity of the compounds due to methylation led to the anticipation that all methylated compounds would elute later. This observation underscores the different interactions between the investigated substances and the chromatographic system during analysis, despite the structural similarity of the investigated steroids.

#### 3.3.2 CID ion mass spectra

Phase II metabolites of EAAS are often analyzed in negative ESI mode due to high ionization efficiency. However, resulting fragments predominantly represent the sulfate or glucuronide moiety and are therefore not suitable for identification. For example, CID ion mass spectra of sulfated EAAS show only one fragment (m/z 97) in negative ionization mode, representing the sulfate group (HSO_4_^−^). Notably, EAAS lacking any additional functional group beside the keto group show very poor ionization in positive ESI mode. Nevertheless, certain phase II metabolites of EAAS have demonstrated acceptable ionization efficiency in positive ESI mode.^10^, ^13^, ^14^ These include steroids featuring a 3‐keto‐4‐ene structure, like the investigated model compounds. Underivatized TG, EG, TS, and ES have acceptable ionization efficiency in positive ESI mode, and therefore obtained CID spectra do not differ greatly from the following CID spectra obtained for methylated substances.

**FIGURE 2:**
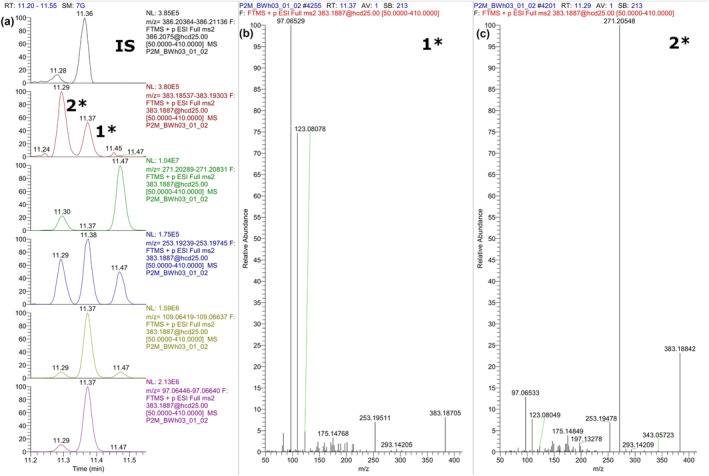
Panel (a) shows extracted chromatograms of methylated TS (1*) and methylated ES (2*) in urine. Panels (b) (1*) and (c) (2*) show the corresponding CID ion mass spectra.

**FIGURE 3:**
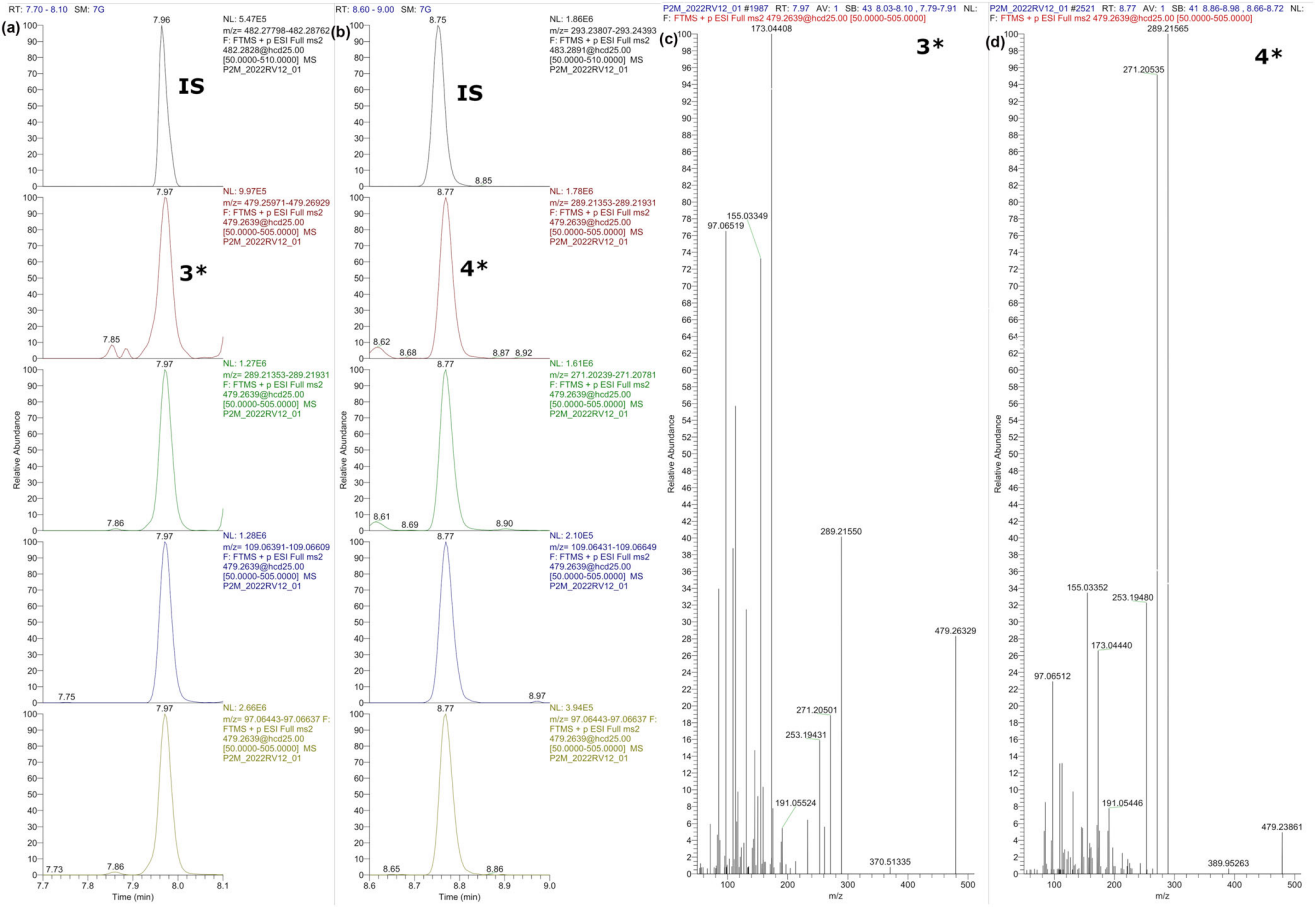
Panels (a) and (b) show extracted chromatograms of methylated TG (3*) and methylated EG (4*) in urine, respectively. Panels (c) (3*) and (d) (4*) show the corresponding CID ion mass spectra.

Methylated conjugated steroids were only analyzed in positive ESI mode, and CID ion mass spectra were investigated for fragments related to the steroid structure. Figure 2 represents the extracted chromatograms and ion mass spectra of methylated TS and methylated ES in urine. Extracted chromatograms and ion mass spectra of methylated TG and methylated EG in urine are shown in Figure 3.

The product ion mass spectrum of methylated TS (Figure 2b) showed two highly abundant ions at m/z 109 and 97 coming from the A ring of the steroid structure.^39^, ^40^ An additional steroid structure relevant ion at m/z 253 was obtained, which corresponds to [M‐SO_3_*‐2H_2_O]^+^. Despite the structural similarity of TS and ES, their CID ion mass spectra differ greatly in ion intensities. The most abundant ion obtained for methylated ES (Figure 2c) was at m/z 271, which corresponds to [M‐SO_3_*‐H_2_O]^+^. Ions at m/z 253, 109, and 97 were also present but at a lower abundance. A similar behavior was observed for the glucuronides, where ions at m/z 109 and 97 were more abundant in methylated TG (Figure 3c) than in methylated EG (Figure 3d). This can be explained by the more unstable bond between E and the conjugated phase II moiety than for T, which has been investigated and described for glucuronides.^33^ This observation also confirms the lower signal obtained for methylated ES in FS mode during method development, which was also explained by lower stability of the substance. The ion at m/z 289 was highly abundant for both ion mass spectra, which corresponds to [M‐Gluc*]^+^. Abundant ions observed at m/z 173 and 155 correspond to the methylated glucuronide ([Gluc*‐H_2_O]^+^, [Gluc*‐2H_2_O]^+^).

The product ion mass spectra obtained in positive ESI mode show more characteristic fragments of the steroid structure than product ion mass spectra obtained in negative ESI mode, which makes them more useful for the identification of steroid conjugates. As mentioned before, it is also possible to obtain product ion mass spectra of underivatized TS, ES, TG, and EG in positive ionization mode, but analysis of methylated conjugated steroids resulted in higher peak intensity and improved peak shape for both glucuronidated and sulfated steroids.

#### 3.3.3 LOQ and linearity

Table 2 summarizes the results obtained for the method parameters linearity and LOQ prepared in artificial urine. Marked transitions were used as quantifiers, for which the LOQ and the linear range were estimated. Other transitions were used as qualifiers.

**TABLE 2:** Estimated method parameters for analysis of methylated conjugated steroids.

Substance	Transitions (m/z)	LOQ (ng/mL)	Linear range (ng/mL)
TG methylated	479.26 → 289.21^a^	0.4	0.4–150
	479.26 → 479.26		
	479.26 → 109.06		
	479.26 → 97.06		
EG methylated	479.26 → 289.21^a^	0.3	0.3–90
	479.26 → 271.20		
	479.26 → 109.06		
	479.26 → 97.06		
TS methylated	383.18 → 97.06^a^	0.2	0.2–60
	383.18 → 383.18		
	383.18 → 253.19		
	383.18 → 109.06		
ES methylated	383.18 → 271.20^a^	X	X
	383.18 → 253.19		
	383.18 → 109.06		
	383.18 → 97.06		

*Note*: X = determination not possible.

^a^ Quantifier.

It was possible to obtain LOQs between 0.2 and 0.4 ng/mL for the methylated substances TS, TG, and EG. Comparing the results to previously conducted experiments without derivatization,^15^ where the corresponding LOQs were calculated between 0.4 and 1 ng/mL, the derivatization method demonstrated a notable improvement, achieving results with a twofold enhancement in LOQ performance. Calibration curves prepared in artificial urine had a coefficient of correlation (*R* ^2^) > 0.99 for the same substances, which indicated excellent linearity within the respective calibration range for each compound. Plots of linear ranges for the substances TS, TG, and EG can be found in the supporting information (Figure S4). However, it was not possible to obtain reproducible signals for ES in the desired concentration range. Despite the structural similarity of TS and ES, significant differences in method characteristics were observed. As already discussed above, conjugated E differs from T regarding stability. The more fragile bond between the steroid and the phase II moiety could contribute to differences in the behavior and therefore different results for method parameters.

#### 3.3.4 Accuracy

To show the applicability of the described methylation method for the quantification of EAAS, calculated values obtained for TG and EG were compared with values obtained from samples of external quality assessment schemes (EQAS). Table 3 shows the mean deviation of concentrations calculated for T and E obtained from methylated TG and EG compared with the assigned concentrations for T and E. The maximal relative standard deviation (RSD) obtained from the determination in triplicates was 12% for TG and 3% for EG.

**TABLE 3:** Quantification of methylated TG and EG and deviation from assigned values of the EQAS samples.

Sample	TG → T (ng/mL)	T assigned (ng/mL)	Deviation T/T assigned	EG → E (ng/mL)	E assigned (ng/mL)	Deviation E/E assigned
Urine 01	24.4	22.3	9.4%	24.3	25.3	−4.1%
Urine 02	21.7	20.7	4.9%	20.7	22.0	−6.0%
Urine 03	33.0	34.4	−3.9%	19.1	23.4	−18.2%
Urine 04	28.9	29.5	−1.9%	69.4	72.0	−3.6%
Urine 05	61.6	63.5	−3.1%	32.5	36.6	−11.2%
Urine 06	22.4	22.1	1.4%	17.4	18.4	−5.2%
Urine 07	36.9	33.0	11.8%	28.1	29.8	−5.7%
Urine 08	63.9	63.8	0.1%	32.4	36.5	−11.3%
Relative mean deviation			4.7%			8.2%

The relative mean deviation for T and E compared with assigned values was determined to be 4.7% and 8.2%, respectively. The target relative standard deviation of 20% (maximum measurement uncertainty for quantification of EAAS according to to the WADA document^8^) was not exceeded by any of the tested samples. Hence, by applying the method described in this article, it was possible to correctly quantify the phase II metabolites TG and EG by LC‐HRMS.

## 4 CONCLUSION

Methylation with TMSD was investigated as a supplementary derivatization technique for the analysis of phase II metabolites of endogenous steroids. Derivatization with 50 μL TMSD and an incubation time of 2 h were found as optimal reaction conditions. Referring to the investigated model compounds (TS, ES, TG, EG), an improvement in peak shape was achieved for glucuronides and, especially, sulfates. Peak widths of 0.06 min measured for all methylated model compounds were at least 2.7 times narrower than peaks measured from underivatized conjugated steroids. It was demonstrated that the peak height increased compared with underivatized steroid conjugates monitoring extracted chromatograms acquired in PRM mode. CID ion mass spectra of methylated, conjugated steroids obtained in positive ESI mode showed structural information regarding the steroid structure, which is useful for identification. To assess the applicability of the results from this preliminary study, selected method performance parameters were determined. Regarding quantitative analysis, linearity and LOQs lower than 0.5 ng/mL could be established for methylated TS, TG, and EG. No reproducible results for concentrations in the desired linear range (<30 ng/mL) could be obtained for methylated ES. These results show that the described derivatization technique is highly dependent on the substance, where minor differences in the structure and stability of the molecule lead to varying results. The applicability of the method for quantification was demonstrated by the comparison of results with assigned values from EQAS samples, showing 4.7% and 8.2% relative mean deviation for TG and EG, respectively. This preliminary study demonstrates the application of the proposed method as a supplementary approach to conventional GC‐MS/MS analysis of unconjugated, endogenous steroids. The improvements in chromatographical behavior due to methylation of the acidic moiety can be beneficial for the development of sensitive methods for the analysis of low abundant phase II metabolites of steroids. To explore the introduced methylation technique further, the next steps will include the analysis of additional phase II metabolites of EAAS including more challenging substances, for example, EAAS lacking any additional functional group beside the keto group. Based on the findings of this study, an optimized and fully validated method applicable for a wider range of phase II metabolites of EAAS can be developed. Methylation as supplementary derivatization technique can be further tested on additional substance groups, for example, phase II metabolites of exogenous steroids.

## Supporting information


**Figure S1:** During methylation of TG (3*) and EG (4*), multiple peaks were obtained for two times methylated (m/z 493.2801) and three times methylated (m/z 507.2958) side products.

**Figure S2:** A shows extracted chromatograms of underivatized TS (1) and ES (2) in urine. B shows extracted chromatograms of methylated TS (1*) and ES (2*) in urine. Peak height and peak width at 5% peak height were measured.

**Figure S3:** A shows extracted chromatograms of underivatized TG (3) and EG (4) in urine. B shows extracted chromatograms of methylated TG (3*) and EG (4*) in urine. Peak height and peak width at 5% peak height were measured.

**Figure S4:** Linear ranges for the methylated substances TG, EG and TS. The coefficient of correlation (R2) was determined and greater than 0.99.

**Data S1.** Supporting Information
