# Supplementary figures and images for: Methylation of phase II metabolites of endogenous anabolic androgenic steroids to improve analytical performance
Source: Drug Test Anal. 2024 Apr 21;17(2):205–15. doi: 10.1002/dta.3694 (PMC11842169; doi:10.1002/dta.3694)

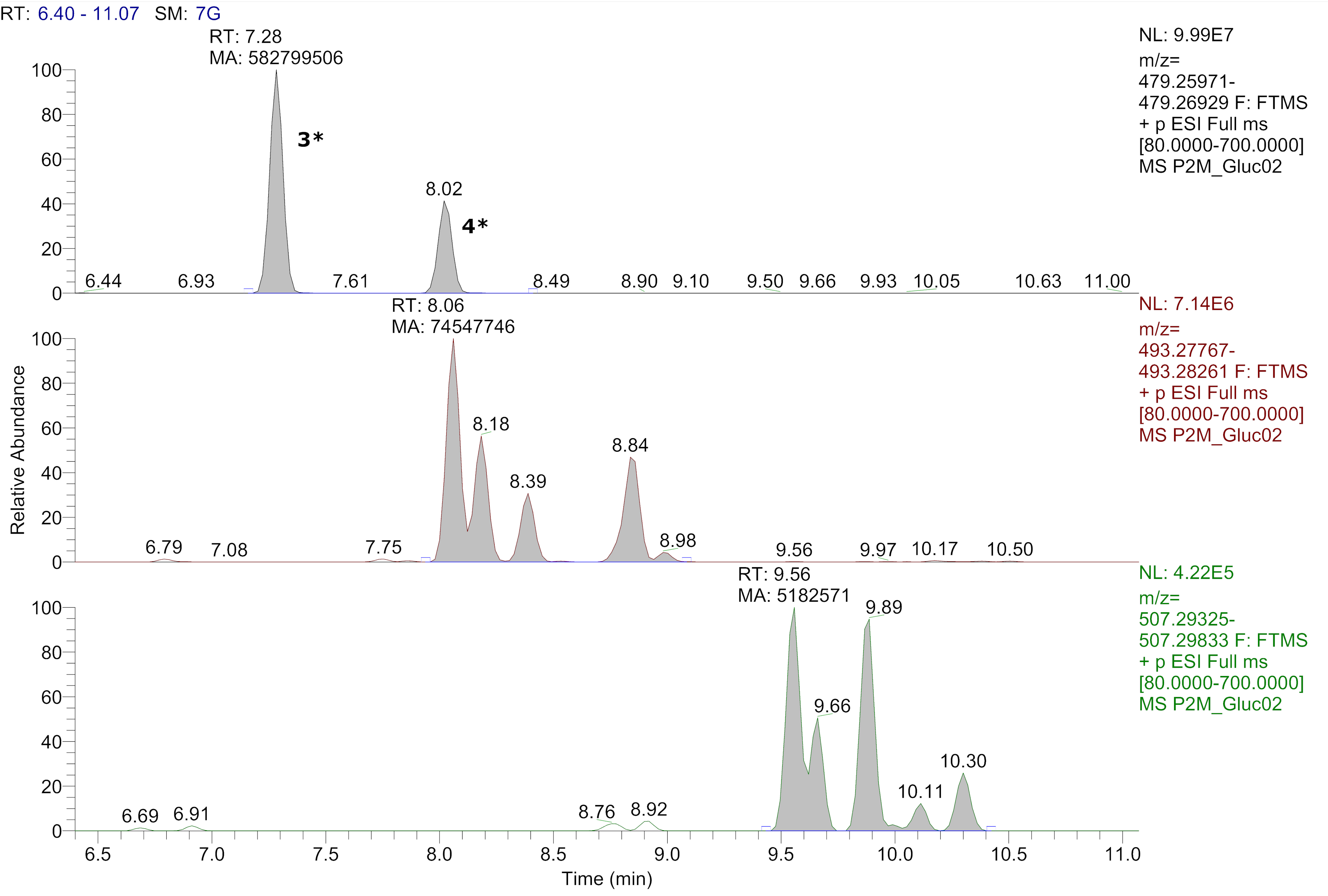

Supplement: Supplementary file 1 — Figure S1: During methylation of TG (3*) and EG (4*), multiple peaks were obtained for two times methylated (m/z 493.2801) and three times methylated (m/z 507.2958) side products. [file DTA-17-205-s004.tiff]

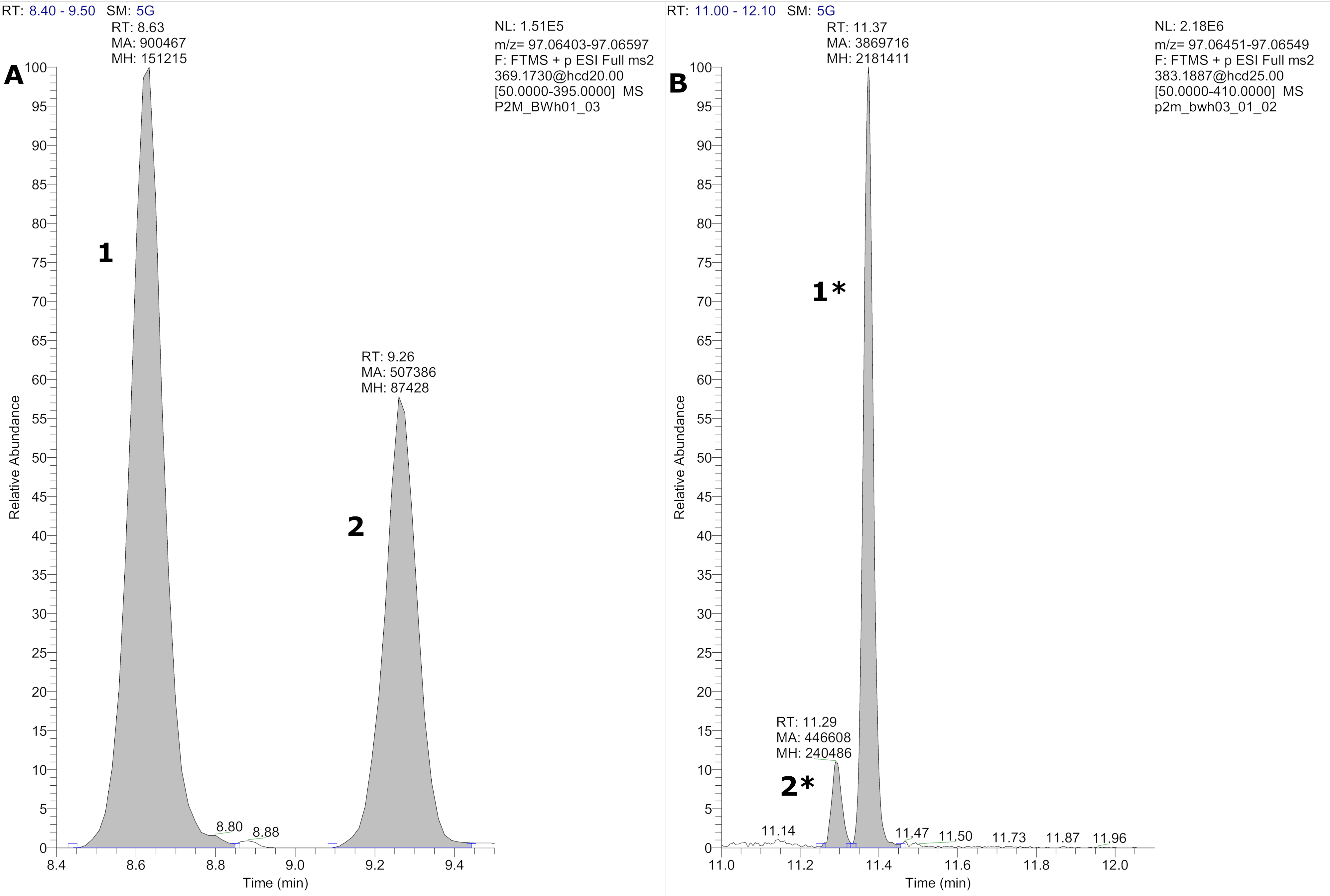

Supplement: Supplementary file 2 — Figure S2: A shows extracted chromatograms of underivatized TS (1) and ES (2) in urine. B shows extracted chromatograms of methylated TS (1*) and ES (2*) in urine. Peak height and peak width at 5% peak height were measured. [file DTA-17-205-s005.tiff]

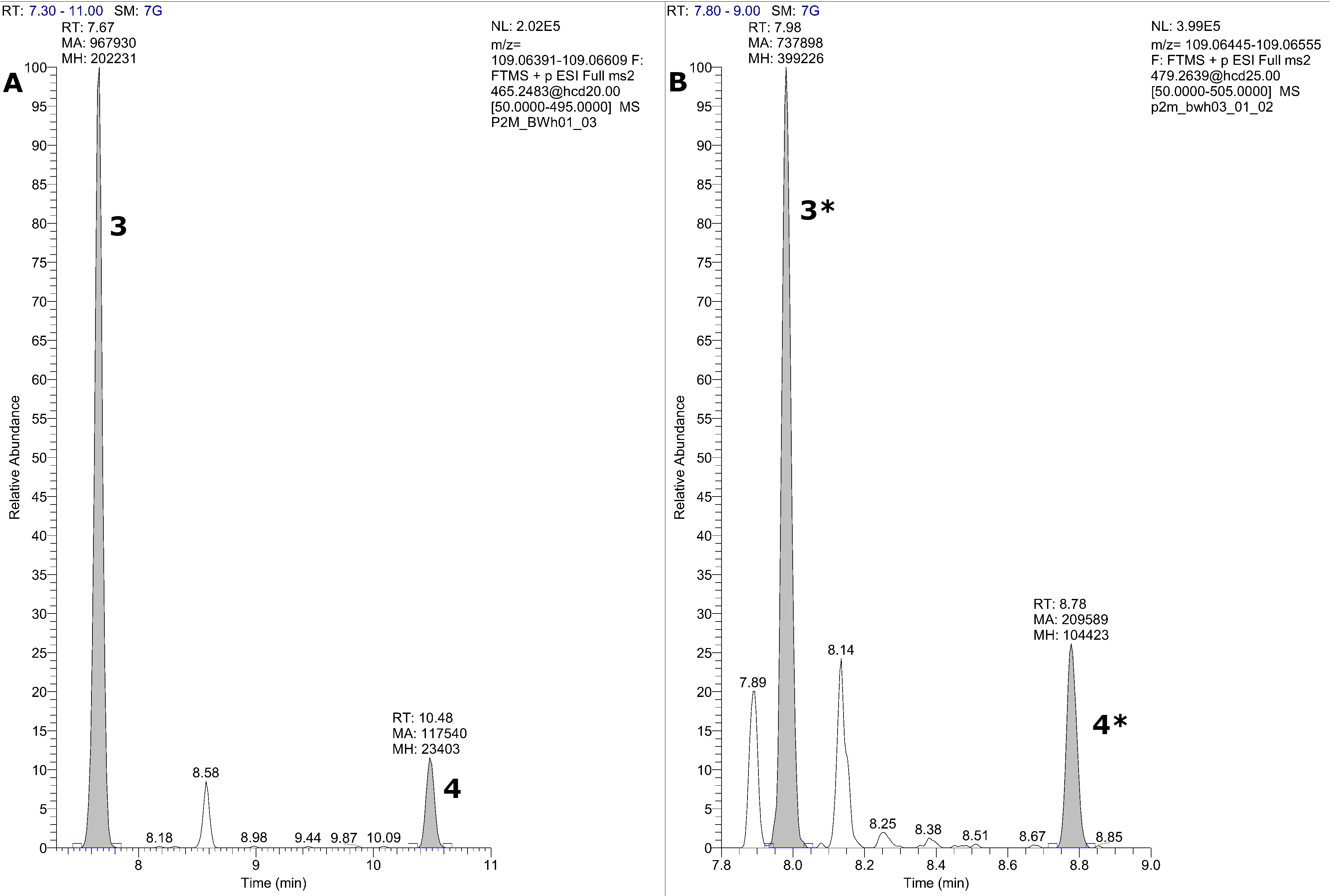

Supplement: Supplementary file 3 — Figure S3: A shows extracted chromatograms of underivatized TG (3) and EG (4) in urine. B shows extracted chromatograms of methylated TG (3*) and EG (4*) in urine. Peak height and peak width at 5% peak height were measured. [file DTA-17-205-s002.tiff]

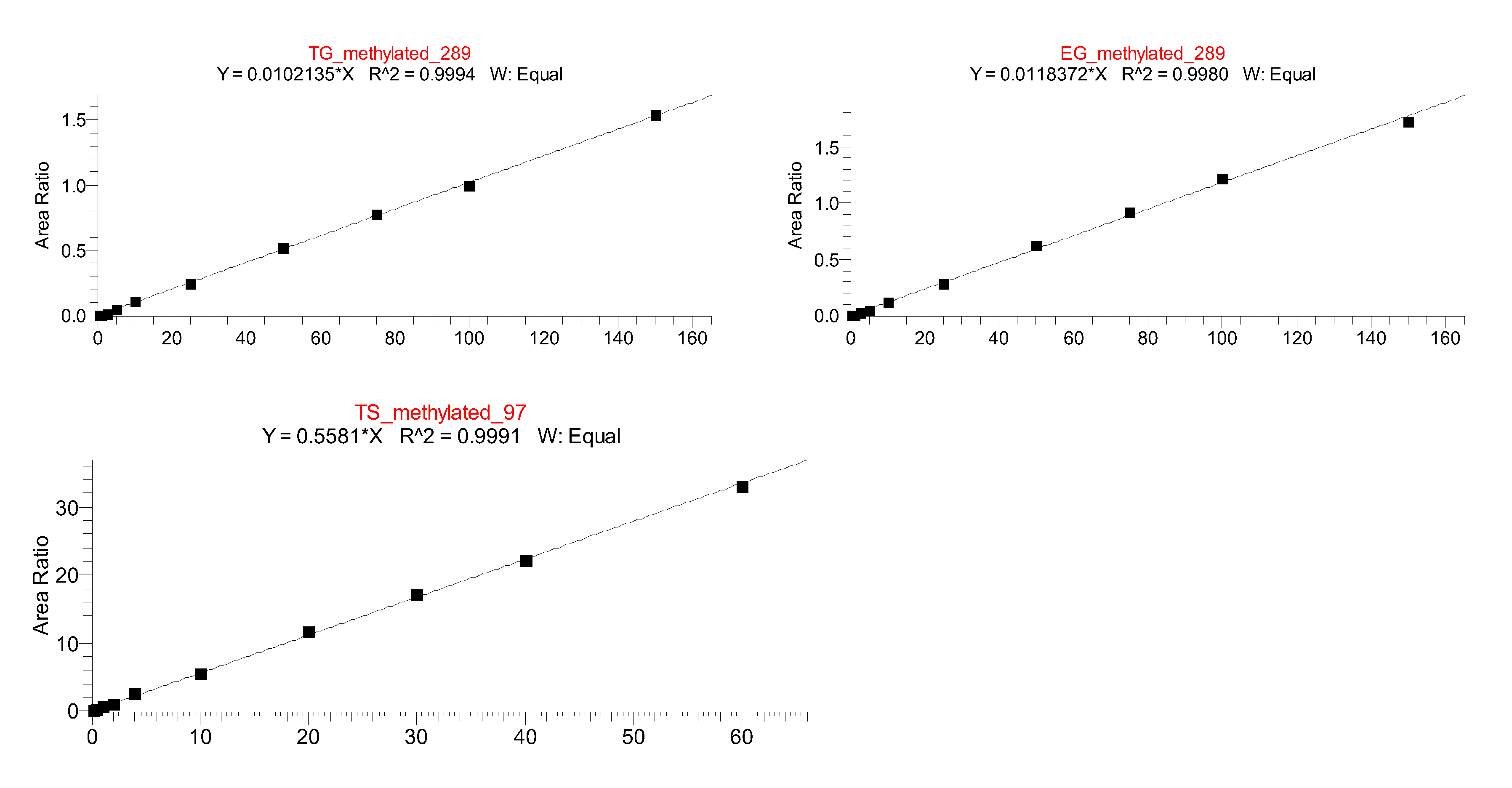

Supplement: Supplementary file 4 — Figure S4: Linear ranges for the methylated substances TG, EG and TS. The coefficient of correlation (R2) was determined and greater than 0.99. [file DTA-17-205-s003.tiff]
