# Supplementary material for: Methylation of phase II metabolites of endogenous anabolic androgenic steroids to improve analytical performance
Source: Drug Test Anal. 2024 Apr 21;17(2):205–15. doi: 10.1002/dta.3694 (PMC11842169; doi:10.1002/dta.3694)
Supplement: Supplementary file 5 — Data S1. Supporting Information [file DTA-17-205-s001.docx]

# Supporting Information


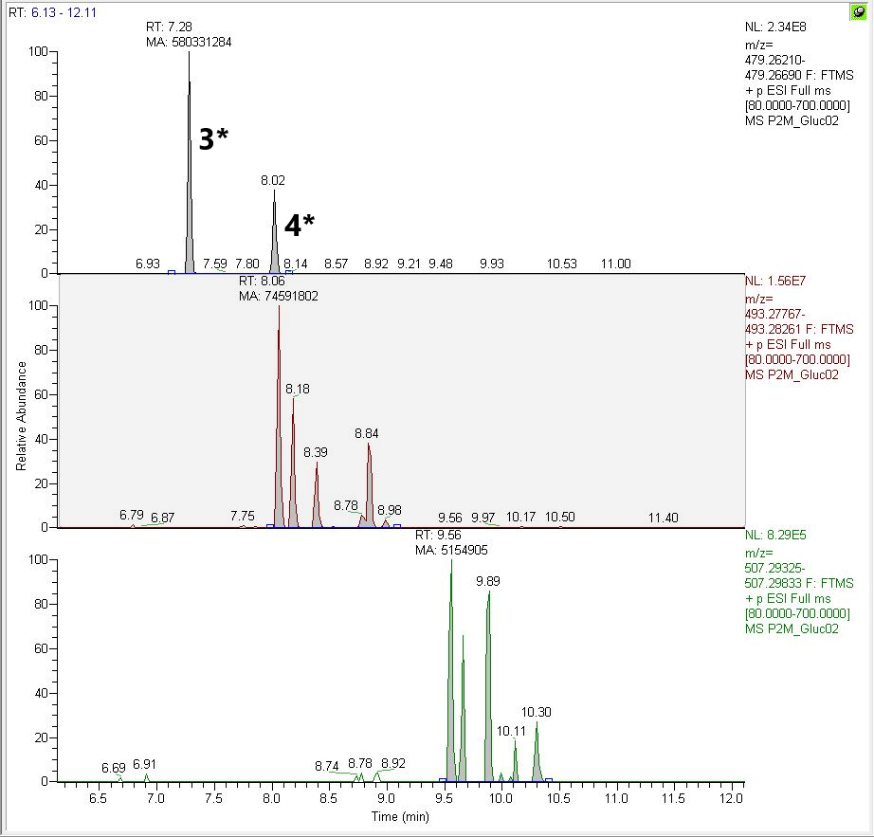


Figure S-1: During methylation of TG (3*) and EG (4*), multiple peaks were obtained for two times methylated (m/z 493.2801) and three times methylated (m/z 507.2958) side products.


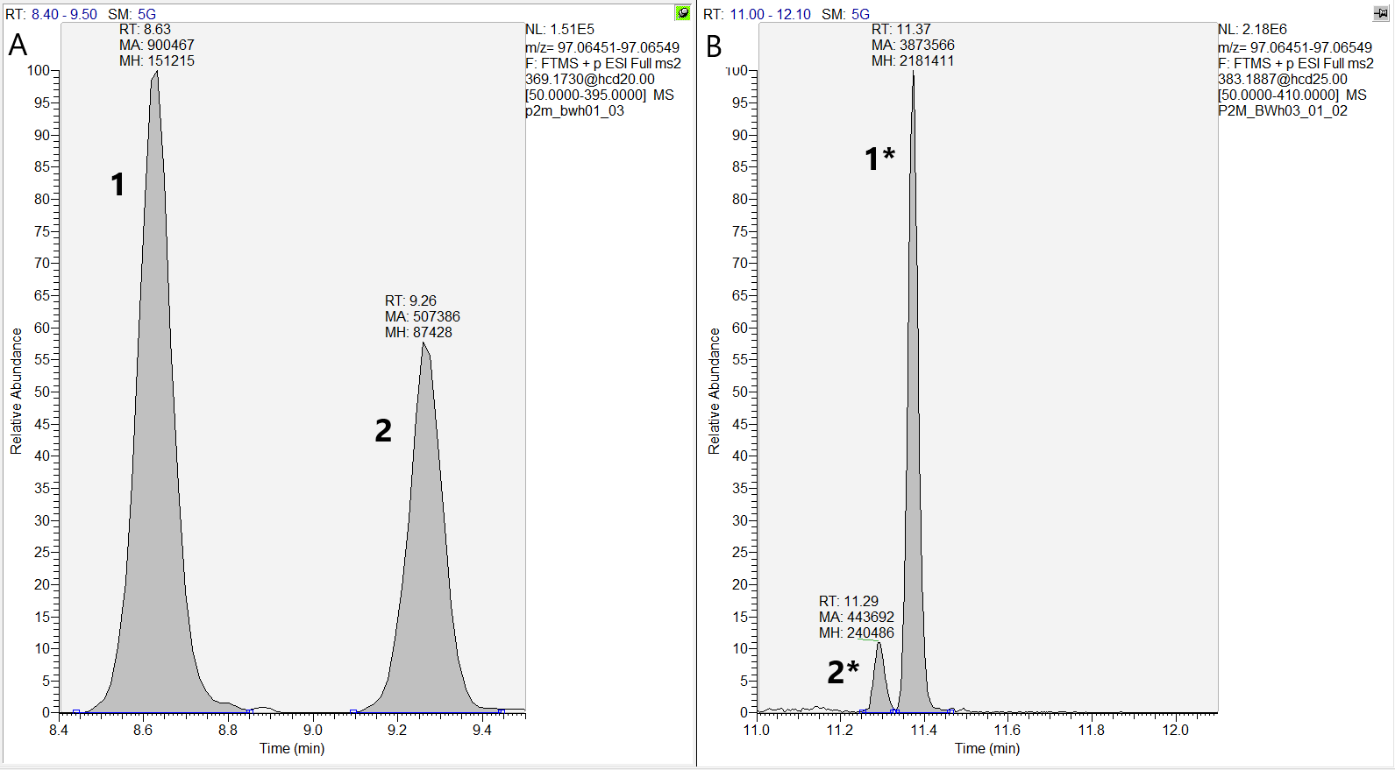


Figure S-2: A shows extracted chromatograms of underivatized TS (1) and ES (2) in urine. B shows extracted chromatograms of methylated TS (1*) and ES (2*) in urine. Peak height and peak width at 5 % peak height were measured.


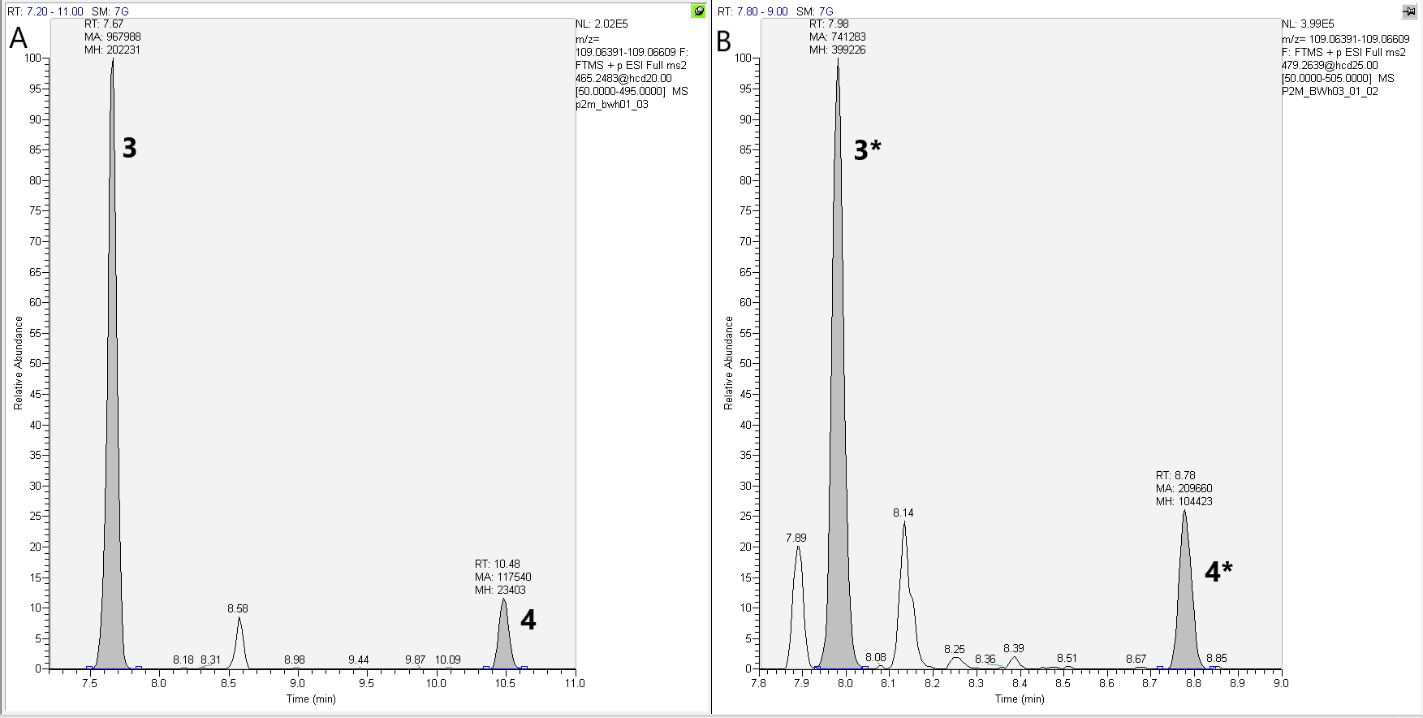


Figure S-3: A shows extracted chromatograms of underivatized TG (3) and EG (4) in urine. B shows extracted chromatograms of methylated TG (3*) and EG (4*) in urine. Peak height and peak width at 5 % peak height were measured.


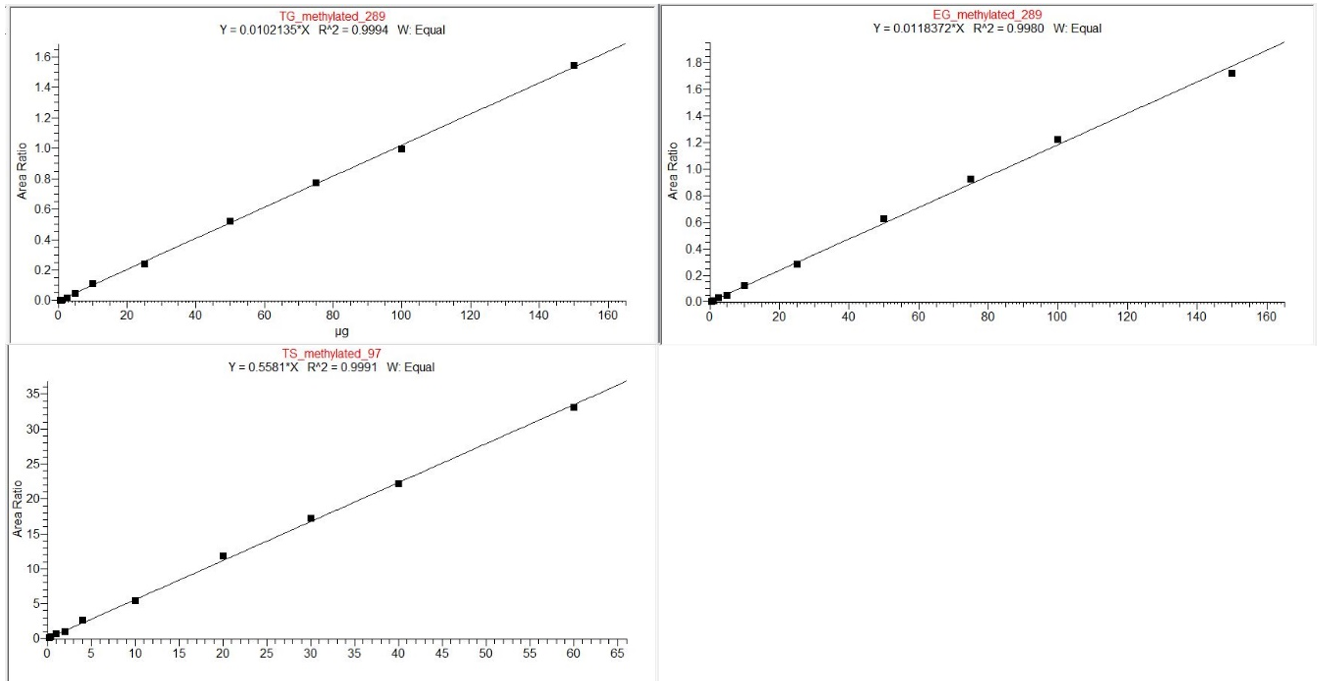


Figure S-4: Linear ranges for the methylated substances TG, EG and TS. The coefficient of correlation (R2) was determined and greater than 0.99.
